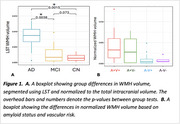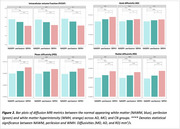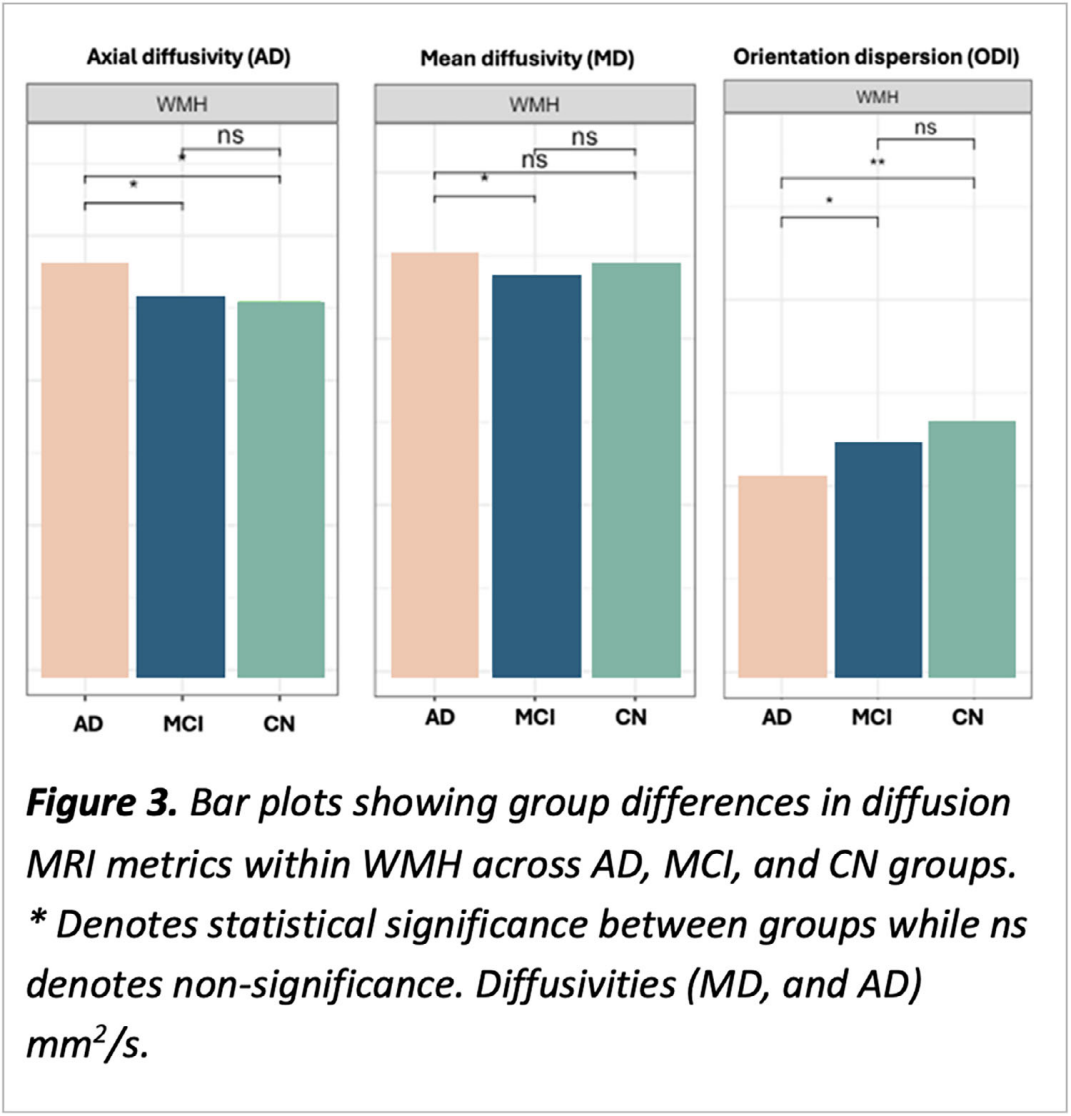# Characterizing White Matter Hyperintensity Pathology in Alzheimer's Disease Using Multimodal Imaging

**DOI:** 10.1002/alz70856_107738

**Published:** 2026-01-09

**Authors:** Yomna Takieldeen, Ho‐Ching Yang, Salman Syed Shahid, Tayyebeh Ebrahimi, Khalid Al‐Ali, Kalen Riley, Brian Graner, Donna M. Wilcock, Andrew J. Saykin, Yu‐Chien Wu, Alzheimer's Disease Neuroimaging Initiative

**Affiliations:** ^1^ Indiana University School of Medicine, Department of Radiology and Imaging Sciences, Indianapolis, IN, USA; ^2^ Center for Neuroimaging, Indiana University School of Medicine, Indianapolis, IN, USA; ^3^ Indiana University School of Medicine, Department of Psychiatry, Indianapolis, IN, USA; ^4^ Indiana University School of Medicine, Department of Neurology, Indianapolis, IN, USA; ^5^ Stark Neurosciences Research Institute, Indiana University School of Medicine, Indianapolis, IN, USA; ^6^ Indiana Alzheimer's Disease Research Center, Indiana University School of Medicine, Indianapolis, IN, USA

## Abstract

**Background:**

White matter hyperintensities (WMHs) in Alzheimer's disease (AD) are often linked to microvascular disease, but emerging evidence suggests AD‐specific pathologies also play a role. This pilot study leverages multimodal imaging to examine WMH volume, microstructure, and perfusion, uncovering distinct vascular and AD‐related contributions through their associations with amyloid and tau.

**Method:**

Thirty cognitively normal (CN), 30 mild cognitive impairment (MCI), and 10 AD participants from the Alzheimer's Disease Neuroimaging Initiative (ADNI3) underwent T1‐weighted, fluid‐attenuated inversion recovery (FLAIR), T2*‐weighted, multi‐shell diffusion MRI, arterial spin labeling (ASL) perfusion, amyloid positron emission tomography (PET), tau PET, and vascular risk assessment. WMH volume was extracted using HyperMapp3r algorithm and Lesion Segmentation Tool (LST), and associated with amyloid and tau burden. To address WMH spatial heterogeneity, imaging metrics were assessed across WMHs, perilesional regions, and adjacent normal appearing white matter (NAWM). Adjacent NAWM was chosen over whole‐brain NAWM to ensure regional comparability within the same white matter tract and reduce intra‐subject variability.

**Result:**

WMH volume was higher in AD compared to MCI and CN (Figure 1A), independent of age, sex, and intracranial volume (F(5, 60)=11.28, *p* <0.001). Amyloid burden (β=0.045, *p* <0.001) was a predictor of WMH volume, independent of vascular risk, while tau was not. When stratifying by amyloid beta status and vascular risk, WMH burden followed a trend where it was highest in amyloid‐positive individuals with high vascular risk (A+V+), followed by amyloid‐positive with low vascular risk (A+V‐), then amyloid‐negative with high vascular risk (A‐V+), and lowest in amyloid‐negative with low vascular risk (A‐V‐) (Figure 1B). Across all groups, WMHs showed the most microstructural damage versus perilesion and NAWM, with a consistent trend across metrics. However, the differences were significant for intracellular volume fraction, axial, radial, and mean diffusivity (Figure 2). Significant between‐group differences were found in orientation dispersion, mean, and axial diffusivity within WMHs (Figure 3).

**Conclusion:**

This study highlights the interplay of vascular and neurodegenerative pathologies in WMH development in AD, with amyloid burden independently contributing to WMH volume and vascular risk amplifying its effects. Future work will examine spatial WMH distribution across amyloid and vascular risk cohorts to clarify their contributions to WMH development and progression.